# Lateral parapatellar approach versus transpatellar splitting approach in intramedullary nailing of diaphyseal tibia fractures, a prospective randomized controlled study

**DOI:** 10.1051/sicotj/2026042

**Published:** 2026-06-18

**Authors:** Amr Amal Amin, El Zaher Hassan El Zaher, Youssef Mohamed Abd-El Salam Wafa, Ahmed Mohamed Khaled El Ghazawy, Amr Mohamed Nagy

**Affiliations:** Orthopedic Surgery Department, Faculty of Medicine, Ain Shams University Cairo Egypt

**Keywords:** Tibial shaft fracture, Intramedullary nailing, Anterior knee pain, Lateral parapatellar approach, Lysholm score

## Abstract

*Background*: Anterior knee pain (AKP) is a frequent complaint after tibial intramedullary nailing (IMN), and the choice of entry approach may contribute to its occurrence. This trial compared the lateral parapatellar semi-extended (LPP) approach versus the midline transpatellar splitting (MLTP) approach for IMN of closed diaphyseal tibial fractures, focusing on AKP and knee function. *Methods*: In this single-blinded randomized controlled trial, 50 adults with acute closed AO/OTA 42 tibial shaft fractures were allocated 1:1 to IMN performed through either the LPP or MLTP approach. Follow-up assessments were performed at 3 and 6 months after surgery. Primary outcomes were AKP during weight-bearing, quantified using a visual analog scale (VAS), and knee function measured by the Lysholm Knee Score. *Results*: LPP achieved higher Lysholm scores at 3 months (82.36 ± 5.2 vs. 75.76 ± 6.1; *P* < 0.001) and 6 months (87.32 ± 4.8 vs. 84.20 ± 5.4; *P* = 0.024), indicating superior early functional recovery. VAS AKP was lower with LPP at 3 months (2.40 ± 0.9 vs. 3.60 ± 1.2; *P* = 0.001), but similar by 6 months (1.80 ± 0.7 vs. 1.96 ± 0.8; *P* = 0.122), suggesting the benefit is mainly early. Operative time favored LPP (62.1 ± 8.7 vs. 68.4 ± 10.2 min; *P* = 0.02) with fewer fluoroscopy shots (29.2 ± 5.4 vs. 38.5 ± 6.1; *P* < 0.001). All fractures united; time to union was comparable (16.8 ± 2.4 vs. 16.2 ± 2.1 weeks; *P* = 0.41). Complications were minor in both groups. *Conclusions*: LPP semi-extended tibial IMN was associated with lower early AKP, better early knee function, and improved operative efficiency without compromising union during 6-month follow-up. A Longer follow-up is required to determine whether anterior knee pain and functional outcomes converge over time.

## Introduction

Tibial shaft fractures are a frequent problem in adult trauma care, most often following high-energy mechanisms such as motor vehicle collisions or falls from height. They commonly affect young, working-age patients and may require lengthy rehabilitation with delayed return to work, which adds a notable socioeconomic burden. Diaphyseal tibial fractures are categorized as AO/OTA type 42, spanning simple, wedge, and complex patterns; this classification helps describe injury severity and informs management decisions [1, 2].

For displaced, closed diaphyseal fractures, intramedullary nailing (IMN) is widely used because it offers load-sharing stability, facilitates early mobilization, and typically results in reliable union with lower infection and malalignment rates than alternative fixation methods [3]. Even so, anterior knee pain (AKP) remains a persistent postoperative concern. Reported incidence varies substantially between studies, which likely reflects differences in follow-up timing, how pain is defined, and variations in technique. The causes are likely multifactorial, including irritation or injury to the patellar tendon, the infrapatellar (Hoffa’s) fat pad, and the infrapatellar branch of the saphenous nerve, in addition to periarticular or intra-articular trauma related to portal creation and nail insertion [4, 5].

The entry portal and the way the surrounding soft tissues are handled are closely linked to these proposed mechanisms of AKP, so the starting approach has been a key target for improvement. In the midline transpatellar (transtendinous) approach, the patellar tendon is split longitudinally to reach the tibial entry point. The lateral parapatellar approach, in contrast, accesses the same start point by retracting the tendon medially, which may reduce direct tendon trauma [6, 7].

Semi-extended nailing through a parapatellar corridor has also gained attention. This position can make fracture reduction and instrumentation easier, with better alignment control, fewer fluoroscopy checks, and a more comfortable early rehabilitation period [8, 9]. Recent comparative studies have further emphasized that the surgical entry approach may influence anterior knee pain, functional recovery, operative efficiency, and radiological outcomes after tibial intramedullary nailing [5, 10, 11].

However, findings across studies are not consistent. It is still uncertain whether the proposed advantages translate reliably into differences in early pain and function, operative efficiency, or radiographic outcomes – especially in randomized designs with standardized perioperative care. Therefore, this study was designed to compare the lateral parapatellar (semi-extended) (LPP) and midline transpatellar splitting (MLTP) approaches for IMN of closed AO/OTA 42 tibial shaft fractures, with the aim of determining their relative effects on AKP and knee function.

## Patients and methods

### Study design and setting

This single-blinded, randomized controlled trial was conducted at Ain Shams University Hospitals between November 2024 and August 2025. Fifty adult patients presenting through the emergency department with acute closed diaphyseal tibial fractures (AO/OTA type 42) were enrolled consecutively and randomized in a 1:1 ratio into two equal groups. The study protocol was approved by the Research Ethics Committee of the Faculty of Medicine, Ain Shams University ([anonymized] 15/2024/2025). Written informed consent was obtained from all participants prior to enrollment and surgery.

### Eligibility criteria

Adults of both sexes aged >18 years with acute closed diaphyseal tibial fractures (AO/OTA type 42) presenting within 2 weeks of injury were eligible. Exclusion criteria were age <18 or >60 years, prior knee surgery, prior ipsilateral ORIF involving the proximal or distal tibia, congenital knee deformity, open tibial diaphyseal fractures, non-ambulatory status, and ipsilateral lower-limb fractures.

### Randomization and blinding

After confirming eligibility, patients were assigned to MLTP or LPP using a computer-generated randomization sequence. Allocation concealment was maintained using sealed, opaque envelopes that were opened in the operating room immediately before incision. Surgeons were necessarily aware of the assigned approach. Postoperative clinical assessments were performed by an investigator who was not involved in the operative steps and was blinded to group allocation.

### Preoperative assessment

Each patient was evaluated on arrival using ATLS principles, with attention to hemodynamic stability and possible associated injuries. We documented the mechanism of injury, time since trauma, relevant comorbidities, and any previous orthopedic procedures. Local examination focused on deformity, swelling, and tenderness, with confirmation that no open wound was present. Neurovascular status was assessed by palpating the dorsalis pedis and posterior tibial pulses and performing motor and sensory examination of the tibial and peroneal nerves.

Imaging included anteroposterior and lateral radiographs of the full tibia, including the knee and ankle. Additional knee or ankle views were obtained when clinically indicated to exclude associated injuries or articular extension. Fractures were classified according to the AO/OTA system (42A–42C). Routine preoperative laboratory workup included a complete blood count, renal function tests, coagulation profile, and blood grouping with cross-matching, followed by anesthetic assessment for operative fitness.

### Surgical procedure

All procedures were performed under spinal anesthesia with strict aseptic technique. A thigh tourniquet was applied. Patients were positioned supine on a radiolucent table with the limb draped free. In the MLTP group, the knee was flexed for infrapatellar access. In the LPP group, the knee was maintained in a semi-extended position (approximately 30° flexion) using a support under the distal femur. Prophylactic cefazolin (1–2 g according to body weight) was administered 30 min before incision.

In the MLTP group, a 3–4 cm midline incision was made centered over the patellar tendon, followed by longitudinal splitting of the tendon to reach the entry portal. In the LPP group, a 3–5 cm incision was made just lateral to the patellar tendon, which was then retracted medially without tendon splitting to expose the starting point. In both groups, the entry point was created under fluoroscopy using a curved awl; on the AP view it was just medial to the lateral tibial spine and slightly medial to the tibial crest, and on the lateral view it was at the anterior edge of the tibial plateau just distal to the articular surface. A ball-tipped guidewire was advanced across the fracture under fluoroscopic control.

Closed reduction was attempted using manual traction under imaging. When required, percutaneous reduction clamps were used without additional incisions. Sequential reaming was performed to 1.0–1.5 mm larger than the selected nail. A titanium tibial intramedullary nail (length 280–380 mm; diameter 9–13 mm) was inserted over the guidewire (Al Markaz Al Dawly^®^). Proximal locking was performed using crossed screws, and distal locking was achieved using two medial-to-lateral interlocking screws using the nail system or free-hand technique under fluoroscopy. No drains were used. Wounds were closed in layers with sterile dressing. Operative time was defined as skin incision to closure.

### Postoperative management and rehabilitation

Patients were monitored in the post-anesthesia care unit and then transferred to the ward, with serial assessment of vital signs, limb neurovascular status, and wound condition. Intravenous cefazolin was continued for 48 h postoperatively. Thromboprophylaxis with enoxaparin 40 mg subcutaneously once daily was started 12 h after surgery and continued for 10–14 days, with duration adjusted to individual risk and mobility. Analgesia followed a multimodal regimen using paracetamol, NSAIDs, and opioids as needed, with pain assessed using a visual analog scale (VAS). Physiotherapy was initiated on postoperative day one, including active and passive knee and ankle range-of-motion exercises. Weight-bearing progression was guided by fracture pattern and fixation stability, with delayed full weight-bearing in comminuted fractures until radiographic progression to union. Discharge occurred once patients were afebrile, ambulatory with aids, and had adequate pain control on oral medication, with standardized instructions for wound care and follow-up.

### Outcome measures and follow-up schedule

Patients were reviewed at 6 weeks, 3 months, and 6 months postoperatively. The primary clinical outcomes were AKP measured by VAS during weight-bearing and functional outcome assessed using the Lysholm Knee Score at 3 and 6 months. Preoperative Lysholm scores were not collected because patients presented with acute fractures, and preoperative functional assessment would mainly reflect acute trauma-related pain and disability rather than baseline knee function.

Secondary outcomes included operative time and intraoperative imaging burden measured by fluoroscopy shot count obtained from C-arm console logs.

Radiographic outcomes included fracture union and alignment. Union was defined as a bridging callus on AP and lateral radiographs. Malreduction was defined as >5° varus/valgus in the coronal plane, >10° procurvatum/recurvatum in the sagittal plane, >10–15° rotational malalignment relative to the contralateral limb, >10 mm shortening, or >50% cortical translation at the fracture site. Complications were recorded throughout follow-up, with predefined events including infection requiring antibiotics and/or surgical intervention and knee stiffness requiring physiotherapy beyond the standard protocol.

### Sample size

The sample size was calculated using G*power software version 3.1.9.7 based on a previous study by Cao et al. [12], who reported a large effect size (*d* = 1.447) of pain score at 3 months between intramedullary nailing via the lateral parapatellar approach versus the infrapatellar approach in the treatment of tibial metaphyseal-diaphyseal junction fractures. The total sample size needed to detect such a difference will be 30 patients (15 per group). The sample size will be increased to 50 patients (25 per group) to compensate for possible loss of follow-up and use of a non-parametric test. Alpha and power were adjusted at 0.05 and 0.95, respectively.

### Statistical methods

Data were entered, cleaned, and analyzed using SPSS version 27 (IBM, Armonk, NY, USA). Quantitative data were assessed for normality using the Shapiro-Wilk test and direct data visualization methods. According to normality, quantitative data were summarized as means and standard deviations or medians and ranges. Categorical data were summarized as numbers and percentages. Quantitative data were compared between the groups using Independent t-test and Mann–Whitney U test for parametric and non-parametric variables, respectively. Categorical variables were compared using the chi-square test, with Fisher’s exact test applied when expected cell counts were small. All analyses were two-tailed, and statistical significance was set at *P* < 0.05.

## Results

**Figure 1:**
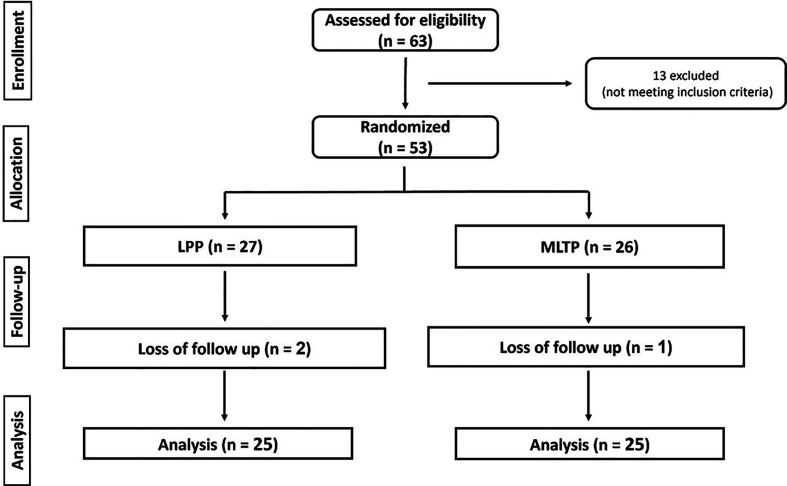
Consort flow diagram of the enrolled patients.

In this randomized controlled trial, 63 patients were assessed for eligibility and 13 were excluded for not meeting the inclusion criteria. The remaining 53 patients were randomized to the lateral parapatellar (LPP) group (*n* = 27) or the midline transpatellar (MLTP) group (*n* = 26). During follow-up, 2 patients in the LPP group and 1 patient in the MLTP group were lost to follow-up. Consequently, 50 patients (25 in each group) completed follow-up and were included in the final analysis (Figure 1).

### General characteristics

Both studied groups were comparable in terms of age (median 28 years [18–46] in the LPP group vs. 29 years [18–55] in the MLTP group; *P* = 0.823) and sex (males: 21/25 [84%] vs. 22/25 [88%]; *P* =1.000).

### Lysholm and VAS scores

Patients in the LPP group had significantly higher Lysholm Knee Scores at both 3 months (82.36 ± 5.2 vs.. 75.76 ± 6.1; *P* < 0.001) and 6 months (87.32 ± 4.8 vs.. 84.20 ± 5.4; *P* = 0.024) compared with the MLTP group. AKP assessed by VAS was significantly lower in the LPP group at 3 months (2.40 ± 0.9 vs. 3.60 ± 1.2; *P* = 0.001), while the difference at 6 months was not statistically significant (1.80 ± 0.7 vs. 1.96 ± 0.8; *P* = 0.122) (Table 1**)**.

**Table 1:** Lysholm and VAS scores between the studied groups.

		LPP (*n* = 25)	MLTP (n = 25)	*P*-value
Lysholm score				
Three months	Mean ± SD	82.36 ± 5.2	75.76 ± 6.1	<0.001*
Six months	Mean ± SD	87.32 ± 4.8	84.20 ± 5.4	0.024*
VAS score				
Three months	Mean ± SD	2.40 ± 0.9	3.60 ± 1.2	<0.001*
Six months	Mean ± SD	1.80 ± 0.7	1.96 ± 0.8	0.122

*n*: number, LPP: Lateral parapatellar approach, MLTP: Midline transpatellar splitting approach, VAS: Visual Analog Scale, SD: Standard deviation, *: Significant *P*-value.

### Intraoperative and radiographic outcomes

Operative time was shorter in the LPP group than in the MLTP group (62.1 ± 8.7 vs. 68.4 ± 10.2 min; *P* = 0.02). The LPP approach was also associated with fewer fluoroscopy shots (29.2 ± 5.4 vs. 38.5 ± 6.1; *P* < 0.001) (Table 2).

**Table 2:** Intraoperative and radiographic outcomes between the studied groups.

		LPP (*n* = 25)	MLTP (*n* = 25)	*P*-value
Operative time (minutes)	Mean ± SD	62.1 ± 8.7	68.4 ± 10.2	0.020*
Fluoroscopy shots	Mean ± SD	29.2 ± 5.4	38.5 ± 6.1	<0.001*

*n*: number, LPP: Lateral parapatellar approach, MLTP: Midline transpatellar splitting approach, SD: Standard deviation, *: Significant *P*-value.

### Fracture union

All fractures in both groups achieved radiographic union within the 6-month follow-up period. The mean time to union was comparable between the LPP and MLTP groups (16.8 ± 2.4 vs. 16.2 ± 2.1 weeks, respectively; *P* = 0.41). No cases of delayed union or nonunion were recorded in either group.

### Complications

Only minor complications were observed. In the MLTP group, two patients (8%) developed superficial wound erythema that resolved with oral antibiotics, and one patient (4%) developed a small superficial hematoma at the incision site that resolved with local measures without further intervention. In the LPP group, one patient (4%) had superficial wound erythema managed conservatively, and one patient (4%) experienced mild transient ankle swelling during early weight-bearing, which resolved with elevation and physiotherapy. No cases of deep infection, implant failure, neurovascular compromise, or reoperation were encountered in either group.

## Cases

### Case 1 (IMN via LPP)

**Figure 2:**
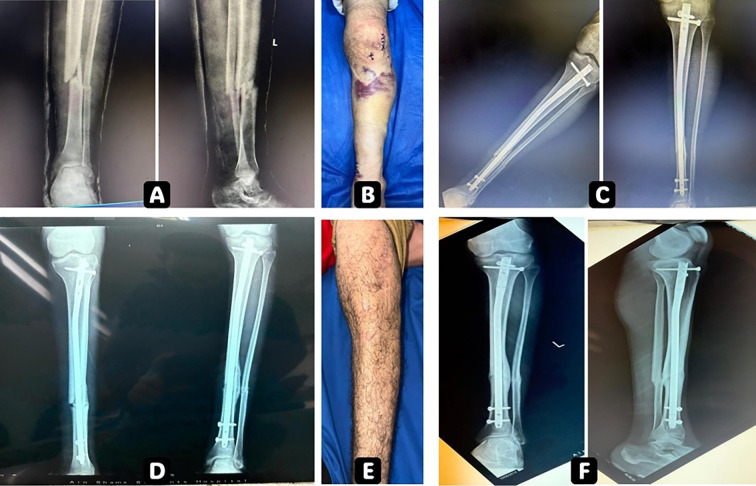
Representative case managed with IMN via the LPP: A) Preoperative anteroposterior and lateral radiographs of a left tibial shaft fracture. B) Clinical photograph showing the first postoperative dressing. C) Immediate postoperative anteroposterior and lateral radiographs. D) Three-month follow-up anteroposterior and lateral radiographs. E) Clinical photograph at 6 months showing the surgical wound. F) Six-month follow-up anteroposterior and lateral radiographs.

An 18-year-old male presented after a road traffic accident with an isolated closed diaphyseal fracture of the left tibia. He had no associated fractures, no relevant comorbidities, and no reported prior knee or leg surgery. Initial assessment included clinical examination with neurovascular evaluation and radiographs of the entire tibia including the knee and ankle. The injury pattern was consistent with a displaced tibial shaft fracture without articular extension. The patient underwent reamed intramedullary tibial nailing using the lateral parapatellar (semi-extended) approach. Standard intraoperative steps were followed, including guidewire passage, sequential reaming, nail insertion, and proximal and distal interlocking. Postoperative care included routine wound management and early initiation of knee and ankle range-of-motion exercises, with progression of weight bearing guided by follow-up assessment (Figures 2A–2F).

### Case 2 (IMN via MLTP)

**Figure 3:**
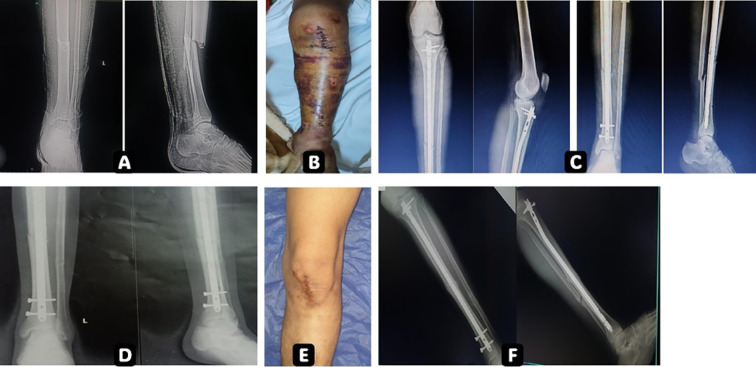
Representative case managed with IMN via MLTP. A) Preoperative anteroposterior and lateral radiographs of a left tibial shaft fracture. B) Clinical photograph showing the first postoperative dressing. C) Immediate postoperative anteroposterior and lateral radiographs. D) Three-month follow-up anteroposterior and lateral radiographs. E) Clinical photograph at 6 months showing the surgical wound. F) Six-month follow-up anteroposterior and lateral radiographs.

A 20-year-old male presented after a road traffic accident with an isolated closed diaphyseal fracture of the left tibia. He had no associated fractures, no relevant comorbidities, and no prior ipsilateral lower-limb surgery. After initial trauma evaluation and documentation of intact distal neurovascular status, radiographs of the entire tibia including the knee and ankle were obtained to define the fracture pattern and exclude articular extension. The patient underwent reamed intramedullary tibial nailing through the MLTP approach with the knee in flexion. Standard technique was followed, including entry portal preparation, guidewire passage, sequential reaming, nail insertion, and proximal and distal interlocking. Postoperative management included routine wound care and initiation of knee and ankle range-of-motion exercises, with progression of weight bearing according to clinical and radiographic follow-up (Figures 3A–3F).

## Discussion

This prospective randomized controlled trial compared the LPP and MLTP approaches for IMN of tibial shaft fractures. The LPP approach was associated with better early postoperative outcomes and greater intraoperative efficiency, while fracture healing was similar between groups. These findings support LPP as a practical alternative to the traditional MLTP portal when early recovery and operative workflow are prioritized.

In our cohort, the LPP group reported less AKP and achieved higher Lysholm scores at the early follow-up points. This aligns with the concept that morbidity after tibial nailing is influenced not only by implant choice and reduction quality, but also by how the extensor mechanism is handled. The question raised by Orfaly et al. [13] – “Does the entry point matter?” – is directly relevant, and our results, alongside current evidence, suggest that it does.

AKP after tibial nailing is multifactorial; however, injury or irritation of the patellar tendon and adjacent anterior knee structures is a plausible contributor. Transtendinous splitting may provoke inflammation in the richly innervated infrapatellar (Hoffa’s) fat pad and can involve the infrapatellar branch of the saphenous nerve [14, 15]. In contrast, the LPP approach allows medial retraction of the tendon without splitting, which may reduce soft-tissue insult. The lower early pain scores in the LPP group are consistent with this rationale and with prior observations supporting semi-extended techniques to limit anterior knee trauma [14]. Although pain scores were similar by 6 months, the early postoperative phase remains clinically relevant because it influences mobility, rehabilitation progress, and patient satisfaction.

Intraoperative metrics also favored the LPP approach. Operative time was modestly shorter and fluoroscopy utilization was lower, with approximately 6 min less operative time and about 9 fewer fluoroscopy shots compared with MLTP. These differences suggest that the semi-extended position used with LPP may facilitate fracture alignment and instrumentation without compromising fixation. Williamson et al. [15] similarly reported reduced radiation exposure with semi-extended nailing, supporting the consistency of this finding. From a technical standpoint, semi-extension may improve reduction by leveraging ligamentotaxis and reducing gastrocnemius-related deforming forces, thereby decreasing reliance on prolonged traction or adjunct reduction maneuvers. In addition, visualization of the tibial plateau on the lateral view to confirm the entry point is often more reproducible in semi-extension than in flexion, which may further explain the reduction in fluoroscopy use.

Despite these approach-related differences in early recovery and efficiency, both techniques achieved the primary goal of fracture surgery. Radiographic healing was reliable in both groups, with comparable time to union and no meaningful difference in overall union rates. This supports the principle that union is driven mainly by biological factors and mechanical stability, provided that the starting point and nail placement are appropriate [13, 16]. In this context, LPP appears to offer early postoperative and intraoperative advantages without sacrificing fracture healing.

Our findings are consistent with reports describing higher rates of AKP after the MLTP approach [14, 17] and with studies noting better early functional scores with parapatellar techniques [18]. Long-term data, such as that of Toivanen et al. [19], which showed no difference in knee pain at 8 years, are not in contrast with results; rather, they suggest that group differences may diminish over time. Even when longer-term outcomes converge, reducing early pain and improving early function remains meaningful to patients and can influence rehabilitation trajectory and return to activity.

Recent evidence also supports these observations. Han et al. [20] found lower early postoperative VAS knee pain with the LPP approach than with a subpatellar approach, with differences present at 24 h, 72 h, and 1 week. They also reported higher scores in several Lysholm subdomains at 12 months – instability, stair climbing, squatting, and pain. Their explanation, that sparing the patellar tendon from splitting and minimizing intra-articular disruption can lessen postoperative knee pain, aligns with the proposed mechanism behind our findings. This supports careful attention to soft-tissue handling when choosing the entry portal for tibial nailing.

Khan et al. [5] reported lower postoperative pain after medial parapatellar nailing compared with the transpatellar tendon approach, supporting the concept that avoiding direct tendon splitting may reduce anterior knee symptoms. Similarly, Sakale et al. compared suprapatellar and infrapatellar nailing in a randomized clinical study and found that approach selection may affect functional and radiological outcomes. Patel et al. reviewed proximal tibial entry points and validated the lateral parapatellar approach as extra-articular, supporting its anatomical rationale and safety profile. These studies support the relevance of our comparison and strengthen the rationale for evaluating the LPP approach as an alternative to MLTP.

This study has several limitations. It was conducted at a single center with a relatively small sample, which may reduce the generalizability of the results. Although the study was powered for the primary outcome, it may not have been sufficiently powered to detect smaller differences in secondary outcomes. Follow-up was limited to 6 months and may not reflect longer-term differences in anterior knee pain or knee function. Preoperative functional scores were not available because of the acute traumatic presentation, which limits direct assessment of within-patient functional recovery from pre-injury status. In addition, surgeons could not be blinded to the assigned approach, which introduces a risk of performance bias. Larger multicenter trials with longer follow-up are needed to confirm these findings and to better characterize longer-term pain and functional outcomes across different patient groups and surgical settings.

## Conclusions

The LPP approach was associated with better early knee function, lower anterior knee pain at 3 months, shorter operative time, and less fluoroscopic exposure than the MLTP approach. However, the difference in anterior knee pain was no longer significant at 6 months, indicating that the pain advantage may be mainly early. These benefits were observed without an apparent trade-off in fracture union during short-term follow-up. Therefore, the LPP approach appears to be a safe and efficient option for tibial shaft intramedullary nailing, although longer follow-up is required before firm conclusions can be made regarding sustained anterior knee pain outcomes.

## Data Availability

Available from the corresponding author upon reasonable request. The data supporting the findings of this study can be obtained upon request from the corresponding author.
